# Study of an arginine- and tryptophan-rich antimicrobial peptide in peri-implantitis

**DOI:** 10.3389/fbioe.2024.1486213

**Published:** 2025-01-07

**Authors:** Qian Zhang, Yalei Jiang, Xiaotong He, Liwei Liu, Xi Zhang

**Affiliations:** ^1^ Department of Periodontology, School and Hospital of Stomotology, Tianjin Key Laboratory of Oral Soft and Hard Tissues Restoration and Regeneration, Tianjin Medical University, Tianjin, China; ^2^ Department of Periodontology, Tianjin Binhai New Area Tanggu Stomatology Hospital, Tianjin, China

**Keywords:** peri-implantitis, antimicrobial peptides, antibacterial activity, wound healing, anti-inflammatory activity

## Abstract

The combination of hydrophilic arginine residues and hydrophobic tryptophan residues is considered to be the first choice for designing short-chain antimicrobial peptides (AMPs) due to their potent antibacterial activity. Based on this, we designed an arginine- and tryptophan-rich short peptide, VR-12. Peri-implantitis is a significant microbial inflammatory disorder characterized by the inflammation of the soft tissues surrounding an implant, which ultimately leads to the progressive resorption of the alveolar bone. This study found through antibacterial experiments, wound healing promotion experiments, and anti-inflammatory experiments that VR-12 inhibited and killed planktonic peri-implantitis-associated bacteria, inhibited biofilm formation, and disrupted mature biofilms. Additionally, VR-12 exhibited good biocompatibility with RAW264.7 cells and human gingival fibroblasts (HGFs) cells, promoting proliferation of both cell types. Moreover, VR-12 induced HGFs migration by promoting expression of migration-related factors, thereby promoting soft tissue healing. VR-12 also acted on lipopolysaccharide (LPS)-induced RAW264.7 cells, exerting excellent anti-inflammatory properties by affecting the secretion/expression of inflammation-related factors/genes. Therefore, VR-12 may be a good option for both warding off and treatmenting peri-implantitis.

## Graphical Abstract

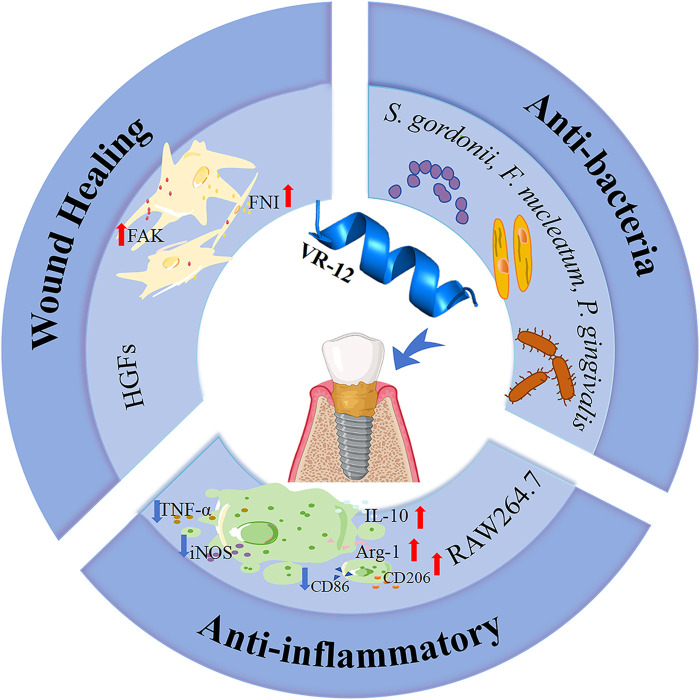

## 1 Introduction

The rising popularity of titanium-finished dental implants for tooth replacement (Chen et al., 2023) has made strategies aimed at preventing and treating peri-implantitis emerge as a significant area of focus. Peri-implant inflammatory processes, including peri-implant hard tissue inflammation and peri-implant mucositis, are initiated by the development of bacterial biofilms, which affect the health of the surrounding soft and hard tissues associated with the implant (Assery et al., 2023). Persistent peri-implantitis can eventually result in implant failure.

Ensuring effective soft tissue sealing around implants is essential for their long-term health and stability (Monje et al., 2023). Additionally, localised application of antimicrobial agents to the “cuff” of the implant can be effective in reducing the incidence of implant inflammation due to adherence of pathogenic bacteria, as well as in promoting the closure of peri-implant soft tissues (Körtvélyessy et al., 2021). Currently, commonly used antimicrobial materials have mainly developed into AMPs and nanomaterials (Dediu et al., 2023; Xing et al., 2023). AMPs, particularly cationic amphiphilic α-helical peptides, are known for their ability to inhibit the growth of microorganisms and biofilm formation directly (Polat et al., 2024). Their unique antimicrobial mechanisms and low risk of resistance development have made them widely utilized in various fields (Rai et al., 2022), making research on AMPs a prominent area in antimicrobial studies (Büyükkiraz and Kesmen, 2022).

In the context of preventing and treating peri-implantitis, pharmaceutical agents should have immunomodulatory properties in addition to antimicrobial and biofilm inhibitory effects (Hancock et al., 2021; Luo and Song, 2021). In addition, these agents should also facilitate the recovery of the peri-implant soft tissues and optimal biological sealing (Garaicoa et al., 2020). AMPs have emerged as a primary focus of our research efforts aimed at the prevention and treatment of peri-implantitis, owing to their remarkable antibacterial efficacy, ability to promote soft tissue healing, and immunomodulatory characteristics (Wessely-Szponder et al., 2019; Xuan et al., 2023).

Host defense peptides (HDPs) not only have excellent antimicrobial properties but also modulate inflammation, stimulate angiogenesis, and facilitate wound healing (Petre, 2020; Drayton et al., 2021; Petkovic et al., 2021). A new synthetic variant of HDPs, called Innate Defense Regulators (IDRs), have antimicrobial, anti-biofilm and immunomodulatory characteristics that confer resistance against various infections (Esposito et al., 2022). IDR-1018 (VRLIVAVRIWRR-NH2) is a cationic antimicrobial peptide containing a pronounced α-helical structure. IDR-1018 demonstrates a broad spectrum of antibacterial and anti-biofilm activities (Wieczorek et al., 2010; de la Fuente-Núñez et al., 2014; Otvos and Ostorhazi, 2015), in addition to its immunomodulatory function of suppressing the generation of tumor necrosis factor-alpha (TNF-a) (Achtman et al., 2012). Notwithstanding this, there are currently no experimental investigations concerning the potential application of IDR-1018 in the treatment of peri-implantitis.

Based on the aforementioned research, a novel short peptide named VR-12 (VRLWVRVRRWRR-NH2) was developed based on IDR-1018. The structural properties of VR-12 were investigated at the beginning of the experiments, followed by the assessment of its antiplanktonic bacterial properties against primary pathogens associated with peri-implantitis and its capacity to impede the formation of biofilms. Additionally, the potential cytotoxicity of VR-12 was also evaluated using HGFs and RAW264.7 murine macrophages to explore its effect on the migratory abilities of HGFs and its anti-inflammatory properties under LPS-induced macrophage inflammatory cell conditions. The primary objective of this research was to evaluate the antimicrobial efficacy of VR-12 while testing its ability to promote soft tissue healing and immune modulation and to determine its suitability for preventing and treating peri-implantitis.

## 2 Materials and methods

### 2.1 Characteristic properties of VR-12

#### 2.1.1 VR-12 preparation

The peptide VR-12 was synthesized by Dechi Biosciences (Shanghai, China). High-performance liquid chromatography (HPLC) analysis indicated 95% purity which was confirmed by mass spectrometry (MS). VR-12 was dissolved and subsequent dilutions of different concentrations were prepared. The basic structural properties of VR-12 were gotten using the program (http://www.pepcalc.com/, accessed 25 September 2023), and helical wheel projections were performed using the program (http://heliquest.ipmc.cnrs.fr/, accessed 25 September 2023). The tertiary structure of VR-12 was analysed using the AlphaFold software (version 3.0; http://golgi.sandbox.google.com/about, accessed 1 September 2023).

#### 2.1.2 Raman spectroscopy

Peptide samples weighing up to 10 mg were subjected to analysis using a Raman spectrometer (Horiba Scientific-LabRAM HR Evolution, Japan). The spectrometer operated with a laser excitation wavelength of 532 nm, an energy resolution limit of 0.43 eV, and an analysis chamber vacuum >5 × 10^−10^ mbar with a test range of 300–4,000 eV.

#### 2.1.3 Circular dichroism (CD) spectroscopy

In order to detect the secondary structure of the VR-12, we concocted a solution of the VR-12 at a concentration of 0.2 mg/mL, using phosphate-buffered saline (PBS) as the solvent. The CD spectra were tested using a CD spectrometer (Jasco-J-1500, Japan). Spectral testing wavelengths ranged from 190 to 350 nm, with a 0.5 nm spectral interval, and a scanning speed of 10 nm/min. Calculated the average molar ellipticity of VR-12, denoted as [θ], in units of degcm^2^·dmol−^1^.

### 2.2 Antibacterial activity experiments

#### 2.2.1 Determination of minimum inhibitory concentration (MIC) and minimum bactericidal concentration (MBC)

*Streptococcus gordonii (*ATCC 10558) is a facultative anaerobic bacterium, *Fusobacterium nucleatum* (ATCC 25586) and *Porphyromonas gingivalis* (BNCC 353909) are obligate anaerobic bacteria. The effectiveness of VR-12 in suppressing bacterial proliferation was assessed using the standard broth dilution method (Tang et al., 2022). Various concentrations of VR-12 (1,000–7.8 μg/mL, serial two-fold dilution) were mixed with liquid medium broth containing bacteria (1 × 10^6^ CFU/mL) at a ratio of 1:1, with an overall volume of 200 µL. A blank control was established using a 200 µL PBS solution, whereas a negative control was prepared by combining 100 µL PBS solution with 100 µL of bacterial culture (1 × 10^6^ CFU/mL). The bacterial cultures were incubated in liquid medium for 24/48 h at 37°C, with daily observation of the bacterial sediment accumulation at the base of the well.

MIC refers to the minimal concentration of peptide that induces a clear liquid and no visible bacterial growth in the well. The bacterial suspension in the well with a concentration greater than the MIC was pipetted evenly and 10 µL of the liquid was extracted from the well and transferred to agar plates and incubated for 24/48 h for MBC determination. The concentration at which no bacterial growth occurs is referred to as the MBC, indicating that the growth of >99.9% of the bacteria was inhibited.

#### 2.2.2 Biofilm inhibition

The impact of VR-12 on biofilm formation was assessed through the measurement of absorbance following crystal violet staining. VR-12 was added to *S. gordonii, P. gingivalis,* and *F. nucleatum* (1 × 10^6^ CFU/mL) and cultured for 24 h (*S. gordonii*) or 48 h (*P. gingivalis, F. nucleatum*). After incubation, the biofilm in each well was subjected to fixation using 95% methanol and subsequently stained with 0.5% (w/v) crystal violet, which was ultimately dissolved in ethanol. The absorbance was measured at OD_600_.

#### 2.2.3 Confocal laser scanning microscopy (CLSM)

VR-12’s impact on mature biofilms was observed using CLSM (FV1000, Olympus, Japan). *Streptococcus gordonii, P. gingivalis* and *F. nucleatum* (1 × 10^6^ CFU/mL) were cultured in confocal culture dishes for biofilm formation. VR-12 (MIC) was added to the dish. The biofilms were analyzed through the application of an acridine orange/ethidium bromide (AO/EB) staining kit. Stained dead (red) and live (green) bacteria in the mature biofilms were observed using CLSM.

#### 2.2.4 Scanning electron microscopy (SEM)

SEM (Gemini300, Carl Zeiss, Jena, Germany) for observation of the effect of VR-12 (MIC) on the morphological features of *S. gordonii, P. gingivalis* and *F. nucleatum* cells. The bacteria (1 × 10^6^ CFU/mL) were cultured with VR-12 (MIC) for 24 h, and the bacterial sediment was obtained by centrifugation. Subsequently, added 2.5% glutaraldehyde solution (v/v) to the sediment and fixed for 1 h at 4°C. Following fixation, the samples were subjected to dehydration with progressively higher concentrations of ethanol (50%, 70%, 80%, 90%, 100%). Each dehydration step was conducted for a duration of 15 min. The bacterial samples were then freeze-dried and sprayed with a layer of gold, images were acquired using SEM.

### 2.3 Biocompatibility

#### 2.3.1 Hemolysis

Evaluation of the hemolytic activity of VR-12 through the measurement of hemoglobin release from erythrocytes of healthy rats (Liu et al., 2021). Initially, the cells underwent three washing cycles with PBS, followed by centrifugation at 1,000 rpm for 10 min at ambient temperature. After removing the supernatant, a suspension of cells at a concentration of 5% (v/v) was prepared using 0.9% NaCl solution (w/v). Subsequently, 100 μL of peptides at varying concentrations (7.81–125 μg/mL, serial two-fold dilution) were combined with 100 μL of the red blood cell suspensions. The mixture underwent incubation at 37°C for a duration of 1.5 h, after which it was subjected to centrifugation at 1,000 rpm for 5 min, and measurement of absorbance at OD_540_.1% Triton X-100 and NaCl (0.9%) were used as positive and negative controls, respectively. The hemolysis rate (HR) was determined by employing this formula:

$$\text{HR}\left(\%\right)=\frac{\text{OD}t-\text{OD}n}{\text{OD}p-\text{OD}n}\times 100\%$$

where OD*p,* OD*n* and OD*t* were the absorbance values of the positive control group, the negative control group and the test group, respectively.

#### 2.3.2 Cell culture

RAW264.7 cells (murine macrophage cell line, ATCC, Manassas, VA, United States) and HGFs (ATCC, Manassas, VA, United States) were cultured (37°C, sterile atmosphere of 5% CO_2_) in DMEM (Gibco, United States) containing 10% (v/v) fetal bovine serum (FBS, Solarbio Co., Ltd., Beijing, China) and 1% penicillin/streptomycin (Gibco, Carlsbad, CA).

#### 2.3.3 Cell proliferation assay

We assessed cell growth using the Cell Counting Kit-8 (CCK-8) assay (Chen et al., 2022). Cells (1,000 cells/well) were seeded in a 96-well plate. After 24 h, the cells were treated with VR-12 (7.81–125 μg/mL, serial two-fold dilution) for 1, 3, 5, and 7 days. The wells without VR-12 were used as blank controls. Cells were incubated using the CCK-8 kit (New Cell&Molecular Biotech Co., Ltd., Suzhou, China), and the absorbance was measured at OD_450_.

#### 2.3.4 Live/dead assay

A total of 5 × 10^4^ cells per well were inoculated into 24-well plates. After 24 h, the cells were exposed to two different types of culture medium: regular medium and medium supplemented with VR-12 (62.5 μg/mL). Following a 24-hour incubation period, the cells were incubated with 200 µL of fresh medium containing calcein AM solution and propidium iodide (PI; Beyotime, Shanghai, China) solution, protected from light for 20 min at room temperature. The observations were conducted using inverted fluorescence microscopy (IX71, Olympus, Japan) and images were captured for documentation purposes.

#### 2.3.5 Cellular morphology

The impact of VR-12 (62.5 μg/mL) on cell morphology was investigated in this study. The cell slides were positioned within a 24-well plate, and the cells (1 × 10^4^ cells/well) were inoculated. Following cell attachment, a culture medium containing VR-12 (62.5 μg/mL) or without VR-12 was added, with a culture medium lacking VR-12 serving as the blank group. After 24 h, the cells underwent fixation with 4% (v/v) paraformaldehyde solution for 30 min at room temperature. Subsequently, the cell membrane was rendered permeable through the application of a 0.5% (v/v) Triton X-100 solution. The actin cytoskeleton was visualized using rhodamine phalloidin, while DAPI was for nuclear staining. The cells were observed and imaged through the use of a CLSM.

### 2.4 Cell migration-related experiments

#### 2.4.1 Wound healing assay

HGFs (5 × 10^4^ cells/well) were kept in a 24-well plate, until complete confluence was attained. A vertical line was obtained by carefully scraping the confluent monolayer of HGFs with a sterile 200 μL pipette tip, thereby establishing a consistent cell-free area within each well. The scratch was rinsed thrice with PBS to remove any remaining cells. The fixed region was marked and then incubated with 2% FBS containing medium and VR-12 (62.5 μg/mL). Cells cultured with a concentration of 2% FBS without VR-12 were used as blank controls. The cell migration of the scratched regions was examined using an inverted microscope (Leica, Germany). Scratch images within the same field of view were captured at 0, 6, 12, 24, and 48 h. Images were analysed and scratch widths determined using Fiji software (ImageJ) to calculate the rate of cell migration.

#### 2.4.2 Transwell migration assay

HGFs (1 × 10^4^ cells) were plated into the upper chamber of a Transwell chamber (Costar; Corning, Inc.). In the upper chamber, 100 µL of DMEM with 2% FBS (v/v) was added, while 500 µL of DMEM containing 2% FBS and VR-12 (62.5 μg/mL) was added to the lower chamber. After a 24-hour incubation time, fixed the HGFs with 4% (v/v) paraformaldehyde for 15 min. Subsequently, removal of HGFs from the upper chamber, PBS was used to rinse the chambers, and the migrated HGFs were stained with 0.5% (v/v) crystal violet for 10 min at room temperature. Cells cultured in 2% FBS (v/v) without VR-12 were used as the blank controls. Five random regions at a magnification of ×100 were selected using a light microscope, and the quantitative analysis of the migrated cell count was conducted utilizing Fiji (ImageJ) software.

#### 2.4.3 RNA isolation and quantitative reverse transcription-polymerase chain reaction (RT-qPCR)

To evaluate the impact of VR-12 on the migration of HGFs, RNA was extracted using a RNA extraction kit (Suzhou Xinsaimei Biotechnology Co., Ltd.) after 24 h of incubation. Subsequently, RNA was converted to cDNA by a reverse transcription kit (Takara Biomedical Technology, Japan) for qPCR analysis to quantify the gene expression levels of cytokines fibronectin Ⅰ (FNⅠ) and focal adhesion kinase (FAK). All data were analyzed using the expression of glyceraldehyde-3-phosphate dehydrogenase (GAPDH) as an internal reference gene. The relative expression ratios were presented using the 2^−ΔΔCT^ method (Yuan et al., 2023), which involves comparing the expression levels of GAPDH and the target gene between untreated and treated cells to determine the expression levels. Primer sequences corresponding to the differentiation markers are presented in Supplementary Table S1 of the supplementary data.

### 2.5 Immune modulating effects of VR-12 peptide on RAW264.7 cells

#### 2.5.1 Reactive oxygen species (ROS) production

The intracellular levels of ROS in RAW264.7 cells that have been stimulated by LPS (Sigma-Aldrich, United States) were determined using the DCFH-DA method (Zheng et al., 2022). RAW264.7 cells (1 × 10^5^ cells/well) were cultured in a 6-well plate and stimulated by LPS (1 μg/mL) for 24 h. Then, they were treated with or without VR-12 (62.5 μg/mL) for 24 h. RAW264.7 cells were subjected to washing with PBS and subsequently treated with DCFH-DA (10 μmol/L) for 45 min at 37°C. Negative and positive controls consisted of culture medium and LPS, respectively. Subsequently, the cells were observed under the gaze of an inverted fluorescence microscope. Finally, the cells were collected and analyzed using a Multi-Function Measuring Instrument (Synergy HT, Synergy HT, United States). The formula for the relative content of ROS is as follows:

$$\text{ROS }\left(\%\right)=\frac{Ft-Fn}{Fp-Fn}\times 100\%$$

where F*p*,F*n*,F*t* and were the fluorescence intensity of the positive control group, the negative control group and the test group, respectively.

#### 2.5.2 RT-qPCR analysis

In order to evaluate the anti-inflammatory property of VR-12, the RAW264.7 cells were treated by LPS (1 μg/mL) for a duration of 24 h. Following this treatment, VR-12 (62.5 μg/mL) was either added to the culture medium. The inflammatory genes that were evaluated comprise TNF-α, inducible nitric oxide synthase (INOS), CD86, interleukin-10 (IL-10), arginase-1 (Arg-1), and CD206. GAPDH transcripts were quantified and used as an internal benchmark. The 2^−ΔΔCT^ method was employed to quantify the expression of each gene. Detailed information regarding the primers corresponding for the differentiation markers can be found in Supplementary Table S2 of the supplementary data.

#### 2.5.3 Enzyme-linked immunosorbent assay (ELISA)

The concentrations of IL-10 and TNF-α (Jamshidy et al., 2021) were mearsued using ELISA kits (Multisciences (Lianke) Biotech, Co., Ltd., China). RAW264.7 cells were stimulated with LPS (1 μg/mL) for 24 h, and then they were treated with or without VR-12 (62.5 μg/mL) for another 24 h. Following this, the RAW264.7 cells and cell culture mediums were centrifuged at 4°C and 1,000 × g for 5 min in order to isolate the cell supernatants. The levels of cytokines were quantified by ELISA.

### 2.6 Statistical analysis

All data are presented as mean ± standard deviation (SD). Statistical analyses were conducted using the Prism 9.0 GraphPad Software (GraphPad Software, Inc., La Jolla, CA, United States) for statistical significance. Comparisons among various groups were assessed by *t*-test or one-way analysis of variance (ANOVA). A *p*-value of <0.05 was indicated statistical significance. Every experiment conducted in this study was performed three times to ensure accuracy and reliability, unless otherwise stated.

## 3 Results

### 3.1 Peptide basic characteristics

#### 3.1.1 Peptide characteristics

**FIGURE 1:**
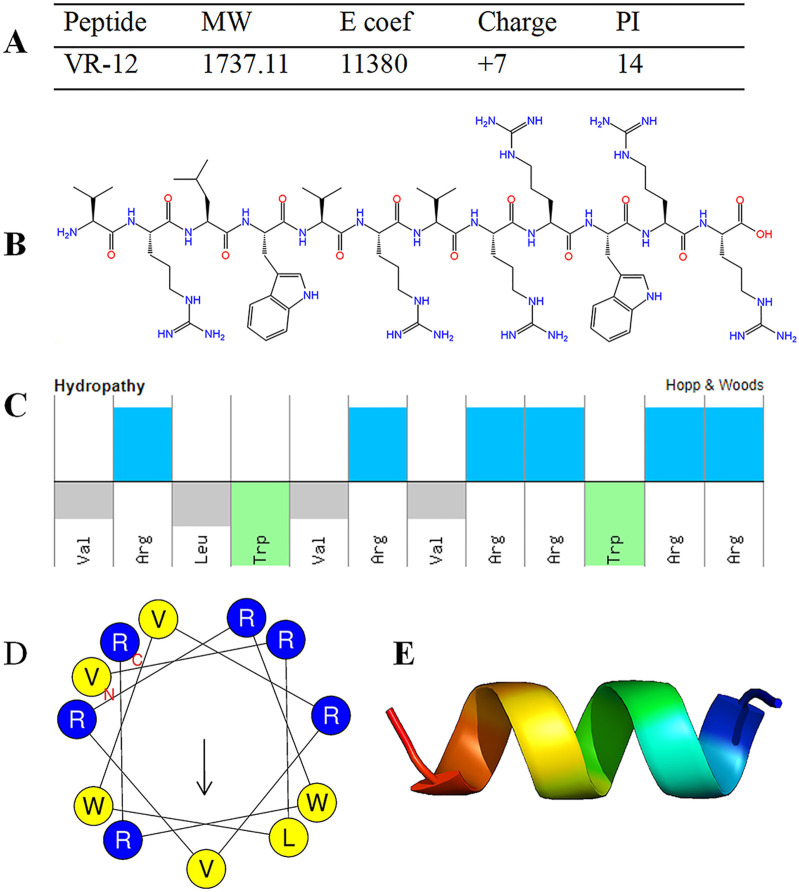
**(A)** Structural parameters of VR-12. **(B)** The molecular formula of VR-12. **(C)** The physicochemical properties and molecular characteristics of the amphiphilic peptide VR-12 (Top: hydrophilic residues; Bottom: hydrophobic residues). **(D)** The helical wheel diagrams of VR-12 (Blue circles: hydrophilic residues; Yellow circles: hydrophobic residues). **(E)** AlphaFold predicts the tertiary structure of VR-12.

The structural characterizations of VR-12 were predicted (Figure 1A). VR-12 demonstrates favorable hydrophilicity, possesses a net positive charge of +7, with a molecular weight of 1737.11 Da in aqueous environments. Figures 1B, C, and D show that the hydrophilic and hydrophobic amino acids within the VR-12 are uniformly distributed along the peptide chain, with 50% of the residues being hydrophobic. Using AlphaFold to assess the tertiary structure of the VR-12, it was observed that VR-12 forms a helical structure, indicating relatively good stability (Figure 1E).

#### 3.1.2 Raman spectroscopy

**FIGURE 2:**
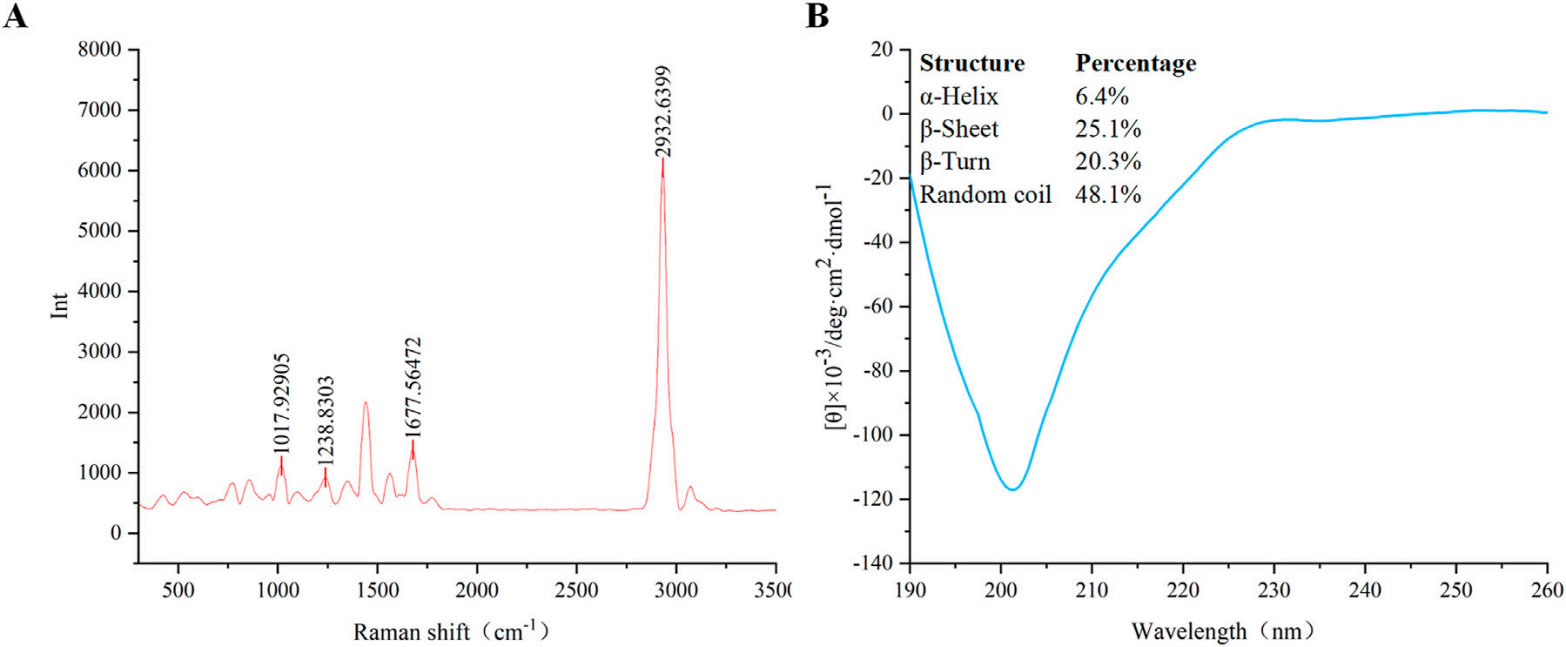
**(A)** Raman spectroscopy of VR-12. **(B)** The CD spectra of VR-12 and CD analysis results for the Secondary Structure of VR-12.

The conformational composition of the secondary structure of AMPs is crucial for their antimicrobial activity. The secondary structure of VR-12 was analyzed using Raman spectroscopy. The wave number range of the Raman spectrum is 500–3,500 cm^−1^ (Figure 2A). Peaks can be seen at 1,018 cm^−1^, 1,239 cm^−1^, 1,678 cm^−1^, 2,933 cm^−1^, representing the skeletal β-helix (Devitt et al., 2019), β-sheet (Lipiec et al., 2021), β turn (Stawoska et al., 2021), CH_2_ and CH_3_ lipid groups (Guleken et al., 2022), respectively.

#### 3.1.3 CD spectroscopy

The proportion of each conformation within the peptide’s secondary structure was analyzed by (Figure 2B). Similar to the results of Raman spectroscopy analysis, VR-12 showed the structure of α-helix, β-sheet, β-turns. The sum of the proportion of α-helix and β-sheet of VR-12 was 31.5%. A higher content of these structures in the secondary structure of AMPs correlated with enhanced stability of the peptides (Ma et al., 2024). Moreover, α-helix and β-sheet structures are crucial for the antimicrobial mechanism of action of AMPs (Bao et al., 2024). Integration of the results from these two analytical techniques indicates that VR-12 not only possesses structural stability but also demonstrates antimicrobial activity.

### 3.2 Antimicrobial activity

#### 3.2.1 VR-12 inhibits different strains growth

The MIC and MBC values of VR-12 for the different bacteria evaluated using the microdilution method are shown in Table 1. These results show that VR-12 exhibits notable antimicrobial efficacy. Among the tested strains, superior antimicrobial efficacy was demonstrated against *S. gordonii.*

**TABLE 1:** MIC and MBC values of VR-12 against *S. gordonii, P. gingivalis* and *F. nucleatum.*

Bacteria	MIC (µg/mL)	MBC (µg/mL)
*S. gordonii*	31.25	62.5
*P. gingivalis*	62.5	125
*F. nucleatum*	62.5	125

#### 3.2.2 Inhibition of biofilm formation

**FIGURE 3:**
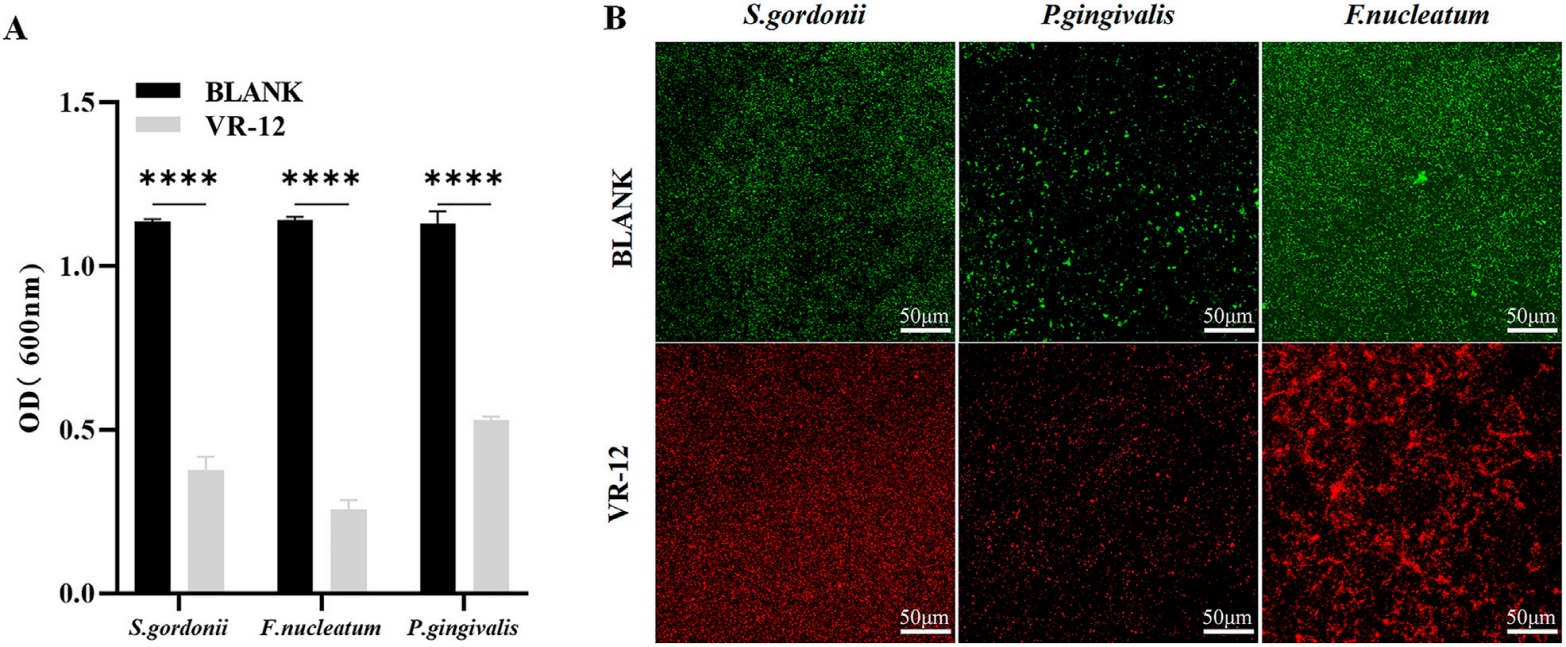
**(A)** Antibacterial biofilm effects of VR-12 against *S. gordonii*, *P. gingivalis*, and *F. nucleatum*. The data were presented as mean ± SD; n = 3, ****p < 0.0001, one-way analysis of variance (ANOVA). **(B)** Bacterial biofilm stained with AO/EB after VR-12 treatment and imaged by CLAM.

VR-12 exhibited enhanced efficacy in inhibiting bacterial biofilm formation, as indicated by significantly lower OD values in VR-12-treated biofilms than that of the blank control (Figure 3A). The confocal laser scanning microscopy (CLSM) images presented in Figure 3B revealed that live bacteria are represented by green dots and dead bacteria by red dots. The blank group exhibited a predominance of green color, indicating denser biofilm formation. In contrast, the VR-12 treated biofilm displayed fewer live bacteria, an increased number of dead bacteria, and a less dense biofilm structure compared to that of the blank group.

#### 3.2.3 SEM

**FIGURE 4:**
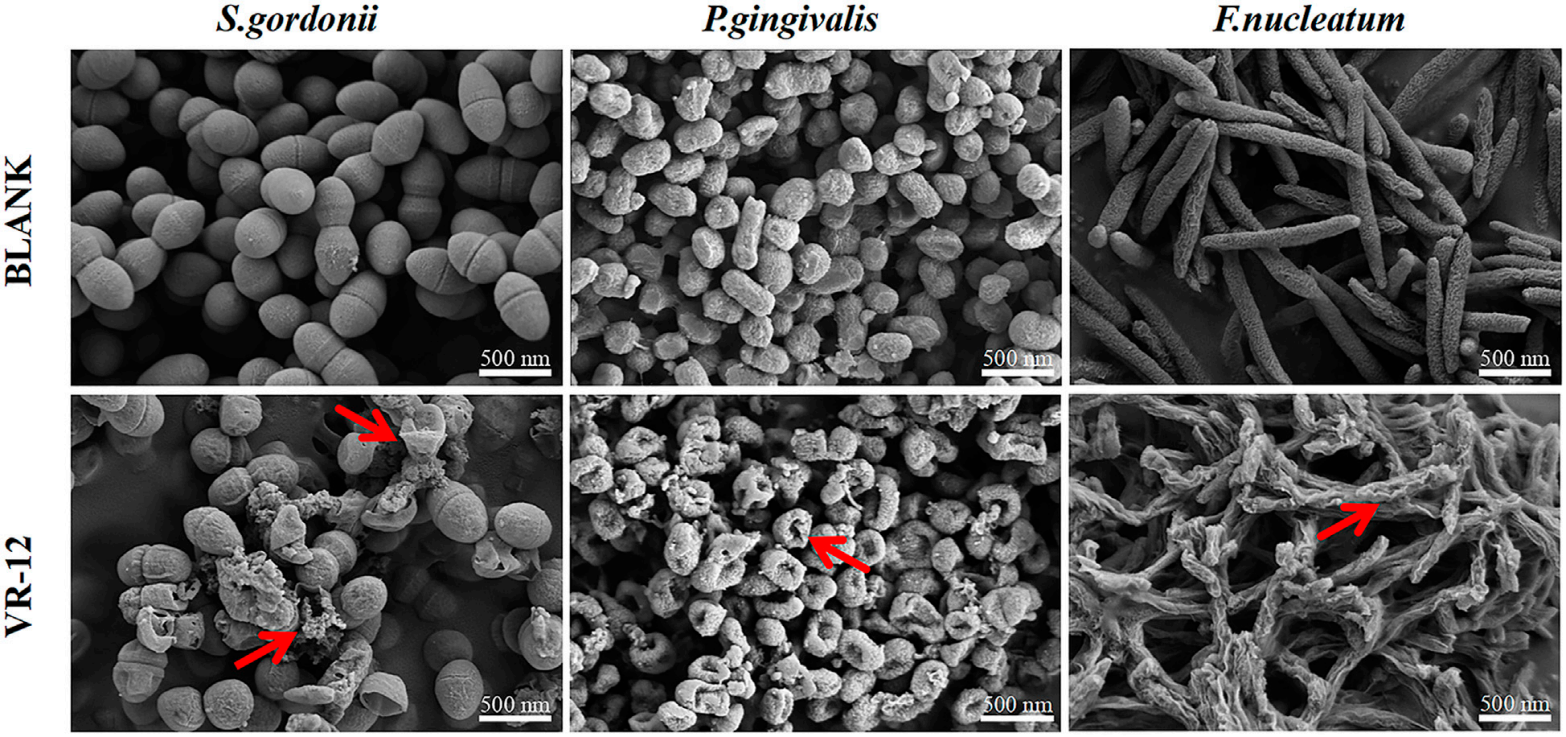
SEM analysis of the effects of VR-12 treatment on *S. gordonii*, *P. gingivalis*, and *F. nucleatum* in comparison to a smooth intact control group revealed significant alterations in the bacterial cell walls after a 24-hour exposure period. These alterations included distortion, corrugation, and damage, as indicated by the red arrows.

The effects of VR-12 on the morphological structure of the three bacterial species were observed using SEM. In contrast to the unaltered and smooth cell surfaces observed in the control group, the bacterial cell membranes treated with VR-12 exhibited noticeable wrinkling, rupture, and leakage of cellular contents. These observations suggested that VR-12 exerted a substantial disruptive effect on the ultrastructure of the bacteria, demonstrating its effective antibacterial properties (Figure 4).

### 3.3 Biocompatibility

#### 3.3.1 Hemolysis

**FIGURE 5:**
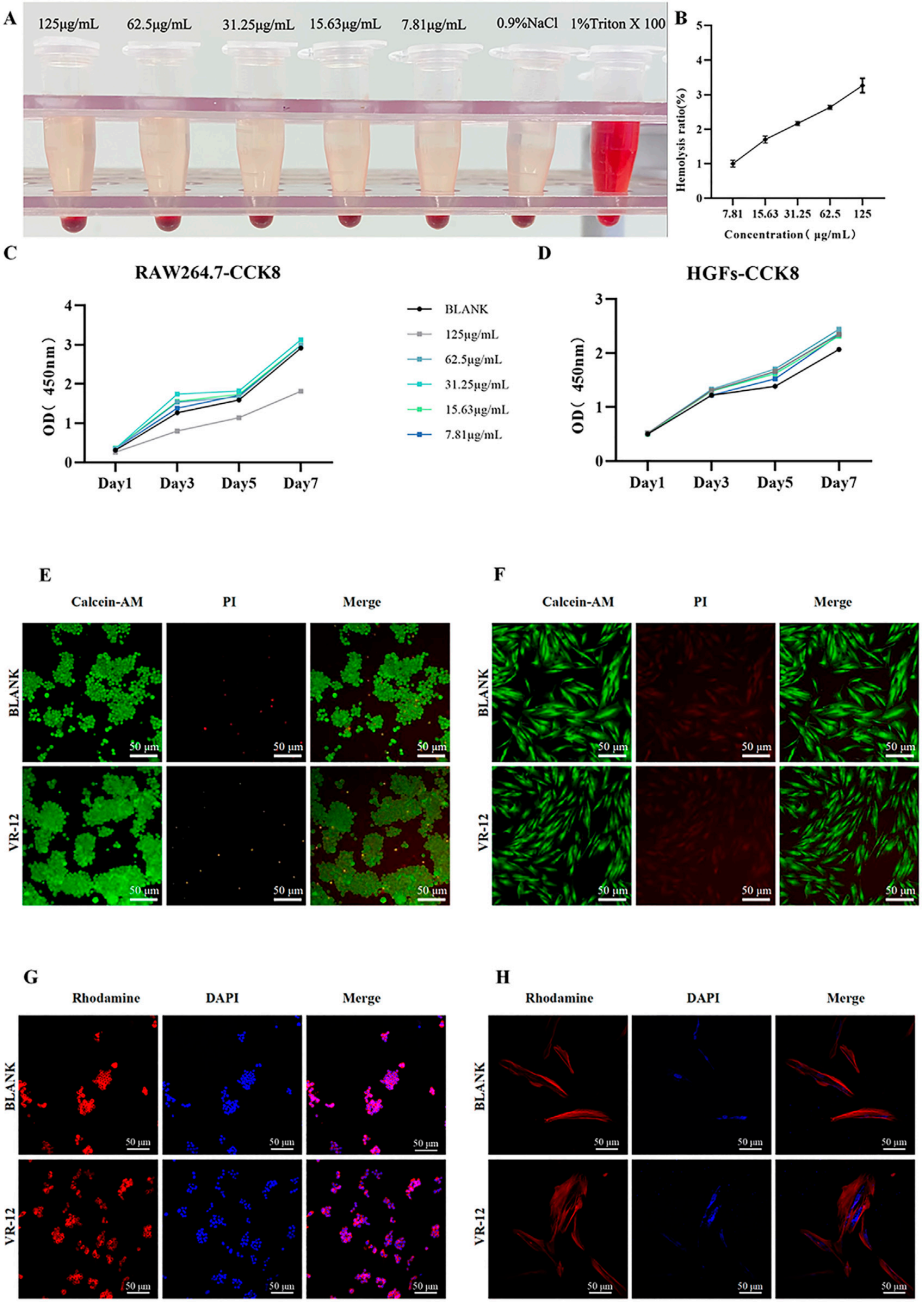
**(A)** Hemolysis Macroscopic Image of VR-12. **(B)** Quantitative results. **(C, D)** CCK-8 results of RAW264.7 cells **(C)**, and HGFs **(D)** after adding VR-12 for 1, 3, 5, and 7 days **(E, F)** After treatment with VR-12 for 24 h, live death of RAW264.7 cells **(E)**, and HGFs **(F)** was observed under a fluorescence inverted microscope using Live/Dead staining. **(G, H)** 24 h after the addition of VR-12, the morphological structure of RAW264.7 cells **(G)**, and HGFs **(H)** was observed under CLSM.

The cytotoxic effect of VR-12 on eukaryotic cells was assessed through its capacity to dissolve rat red blood cells. In Figures 5A, B, it was demonstrated that at the maximum concentration examined (125 μg/mL), the hemolysis rate of VR-12 is 3.27% (*p* < 0.05), which meets the international standard of bio-matters with a hemolysis rate below 5% (Li et al., 2023). The results indicate that VR-12 does not damage the integrity of erythrocytes at effective concentrations and exhibits favorable hemocompatibility.

#### 3.3.2 Cell viability and proliferation

The cell compatibility of VR-12 was confirmed using a CCK-8 assay. Notably, VR-12 exhibited a more pronounced proliferation-enhancing effect on HGFs, with the concentration of 62.5 μg/mL yielding the most significant proliferation promotion within a 7-day period. In RAW264.7 cells, a significant decrease in cell survival was observed when the concentration of VR-12 reached 125 μg/mL, resulting in an inhibition rate of approximately 50%. Conversely, at concentrations below 125 μg/mL, the peptide demonstrated a stimulatory impact on cellular proliferation. These findings suggest that VR-12 displays biocompatibility and has the potential to enhance the proliferation of both HGFs and RAW264.7, at specific concentrations (*p* < 0.05) (Figures 5C, D).

As shown in Figures 5E, F, there was no significant difference in cell viability between the group of cells treated with VR-12 and the group of untreated cells as analysed by live/dead staining. In addition, the number of cells in the designated observation area was significantly increased in the group receiving VR-12 treatment compared to the blank group.

The CLSM images revealed that following a 24-hour treatment with VR-12, the cellular structure remains intact without notable distortion (Figures 5G, H). Additionally, compared with the blank group, HGFs treated with VR-12 exhibited increased cellular adhesion and clearer nuclear morphology. Consequently, the concentration of VR-12 used in the cellular experiments was unlikely to exert a substantial influence on cell morphology and structure.

### 3.4 Cell migration-related experiments

#### 3.4.1 VR-12 promoted cell migration in the wound healing assay

**FIGURE 6:**
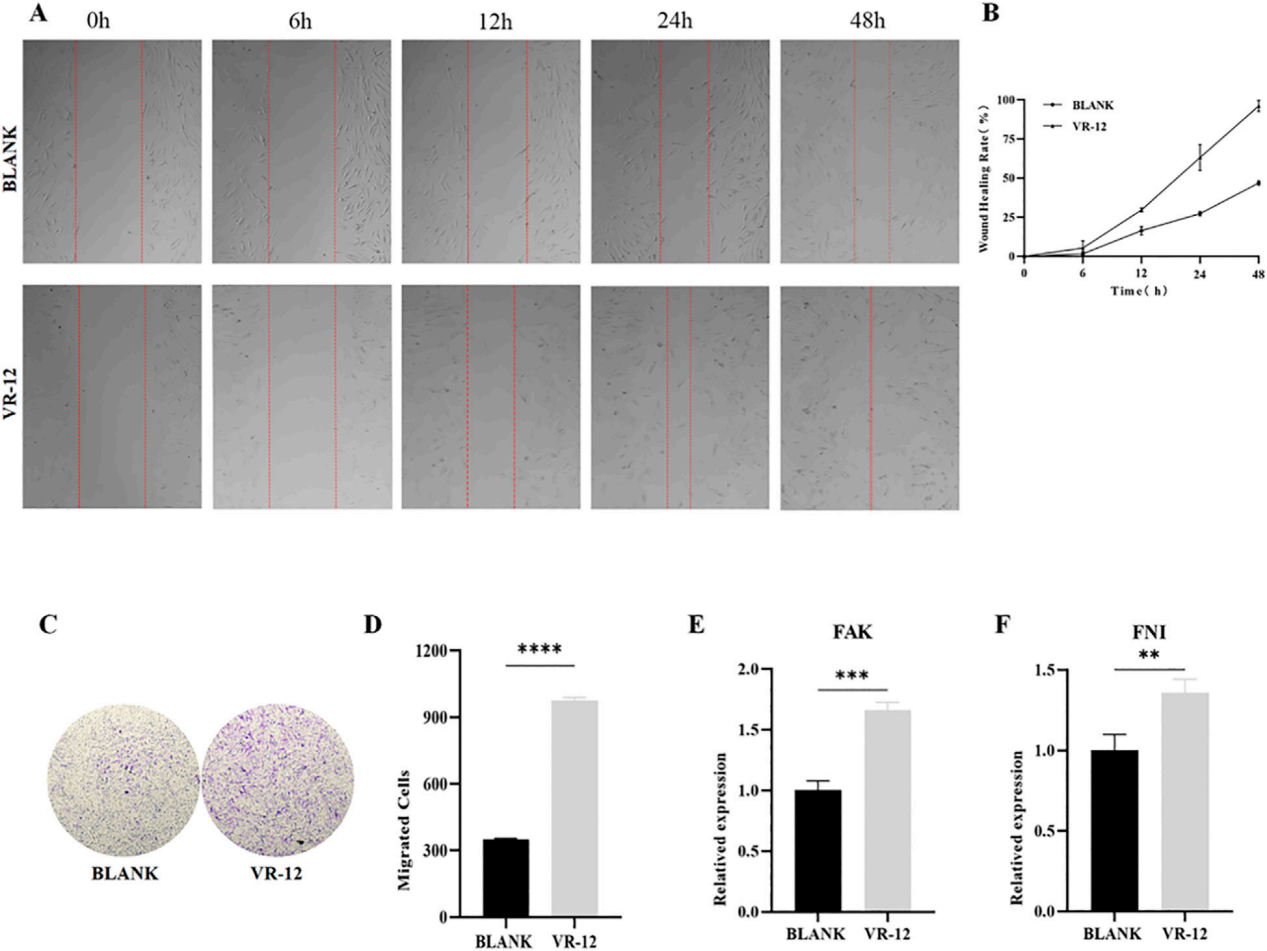
**(A)** Scratch test on HGFs treated with VR-12. The blank group consists of untreated cells. **(B)** Quantitative analysis of the migration rates in **(A, C)** Cell migration of HGFs after treatment with VR-12 was determined by using a transwell migration assay. **(D)** Quantitative analysis of cells that have undergone migration in **(C)**. FAK **(E)** and FNI **(F)** expression of HGFs treated with VR-12 for 24 h were detected by qRT-PCR analysis. All values shown represent mean ± SD; n = 3, **p < 0.01, ***p < 0.001, ****p < 0.0001, ANOVA.

The VR-12 showed a significant promotion of HGFs migration compared to the blank group after 12, 24, and 48 h of culture. Specifically, a marked enhancement in cell migration was observed after 12 h of culture. Moreover, after 48 h of culture, VR-12-treated cells promoted complete wound healing (Figures 6A, B).

#### 3.4.2 VR-12 promoted cell migration in the transwell cell migration assay

The presence of VR-12 in the culture medium notably elevated the number of HGFs traversing the chamber base within 24 h, with a significantly higher count observed compared to the control group. The transwell assay provided additional evidence indicating that the migratory ability of HGFs treated with VR-12 was markedly enhanced compared to that of untreated control cells (*p* < 0.05) (Figures 6C, D).

#### 3.4.3 VR-12 promoted the expression level of migration-related markers

RT-qPCR analysis demonstrated significant upregulation in the expression of the migration-promoting cytokines FAK and FNI in HGFs following the application of VR-12 (Figures 6E, F).

### 3.5 The immune regulatory effect of VR-12

#### 3.5.1 VR-12 inhibited the release of ROS induced by LPS

**FIGURE 7:**
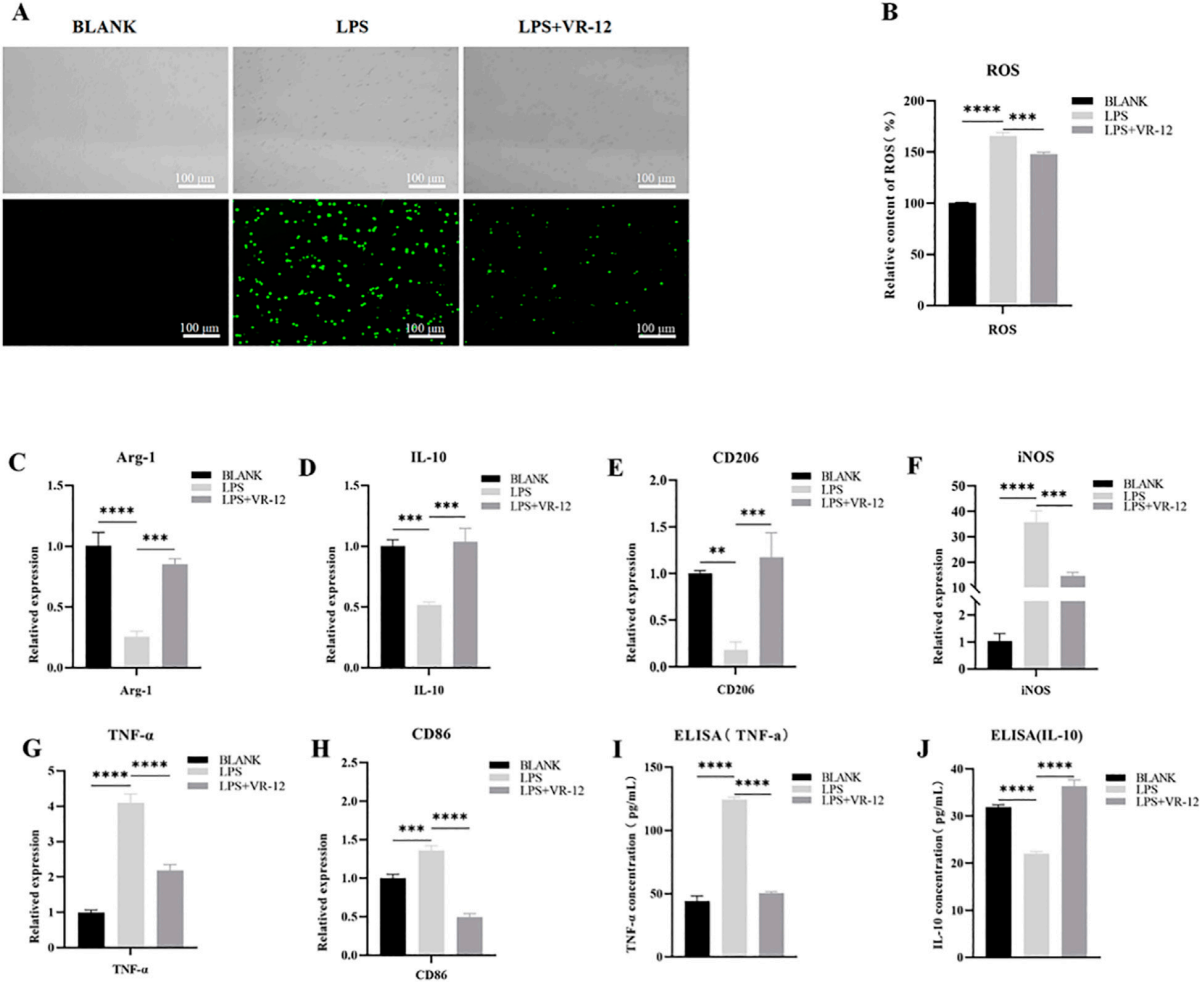
In the context of LPS-induced inflammation, RAW264.7 cells were cultured with VR-12 or culture medium for an additional 24 h. Following this ROS activity was detected using DCFH-DA assay, the alterations in the expression levels of genes associated with inflammation were evaluated using RT-qPCR, and the concentration of IL-10 and TNF-α in the culture supernatant was measured using ELISA assay **(A)** Effect of VR-12 on LPS-stimulated ROS in RAW264.7 cells tested by DCFH-DA method, fluorescence microscopy was used to take pictures **(B)** Quantification of the relative content of ROS in RAW264.7 cells **(C–E)** Expression levels of anti-inflammatory genes in RAW264.7 cells **(F–H)** Expression levels of pro-inflammatory genes in RAW264.7 cells. Effect of VR-12 on TNF-α **(I)** and IL-10 **(J)** contents in RAW264.7 cells. All values shown are means ± SD; n = 3 **p < 0.01, ***p < 0.001, ****p < 0.0001, ANOVA.

To assess the potential of VR-12 in mitigating ROS levels, the DCFH-DA assay was conducted. The results revealed a marked increase in ROS fluorescence intensity in the LPS group compared with that in the control group (Figures 7A, B). VR-12 significantly reduces LPS-induced ROS production. The results of this study suggest that VR-12 can decrease oxidative stress caused by LPS, thereby restoring cells to a normal cellular state.

#### 3.5.2 Effect of VR-12 on the production/gene expression of inflammation-related cytokine in Lps-induced RAW264.7 cells

LPS significantly reduced the mRNA expression of Arg-1, IL-10, and CD206 compared with the blank group (Figures 7C–E). Conversely, VR-12 significantly promoted the transcription of these anti-inflammatory genes. Meanwhile, based on the mRNA expression of pro-inflammatory genes induced by LPS, VR-12 down-regulated the transcript levels of pro-inflammatory-related genes such as INOS, TNF-α, and CD86 (Figures 7F–H). Simultaneously, the secretion levels of TNF-α and IL-10 were assessed using ELISA. VR-12 inhibited the production of the pro-inflammatory cytokine TNF-α (Figure 7I), as evidenced by the significant downregulation of TNF-α gene expression (Figure 7G). Moreover, VR-12 increased the concentration of the anti-inflammatory cytokine IL-10 in LPS-stimulated RAW264.7 cells (Figure 7J), which was attributed to the increased expression of the IL-10 gene (Figure 7D). The results of the ELISA analysis were consistent with those obtained from RT-qPCR analysis. RT-qPCR and ELISA demonstrated that VR-12 modulates the pro-inflammatory effects induced by LPS and exhibits immunomodulatory properties.

## 4 Discussion

At the 2017 World Workshop on the Classification of Periodontal and Peri-Implant Diseases and Conditions, the peri-implant diseases were mainly classified as peri-implantitis, peri-implant mucositis, and peri-implant soft and hard tissue defects (Berglundh et al., 2018). Among them, the main features of peri-implantitis are inflammation of the mucosal tissue surrounding the implant and a slow but steady erosion of the bone that provides essential support (Sun et al., 2023). Within a span of 5–10 years after getting dental implants, the likelihood of developing peri-implantitis can reach to as high as 10% (Onclin et al., 2022). Plaque biofilm is the initiating factor for the development of peri-implantitis, which, if not promptly addressed, can result in alveolar bone resorption and potential failure of the dental implant. Therefore, the removal of bacterial biofilms surrounding implants is crucial for successful implant placement (Martins et al., 2022).

The IDR-1018 peptide is derived from bovine cathelicidin (Bac2a, RLARIVVIRVAR-NH2) (Mansour et al., 2015). By processing and modifying its amino acid sequence, using tryptophan instead of the fourth isoleucine, tryptophan shows a clear preference for the interfacial region of the lipid membrane and has a better penetration effect for the lipid bilayer. Tryptophan-rich AMPs have a stronger membrane-penetrating ability (Khemaissa et al., 2022). Additionally, replacing the sixth alanine and ninth isoleucine with arginine introduces cationic charge and hydrogen bonding capabilities, enabling interaction with anions in bacterial membranes and enhancing antimicrobial activity (Peguda et al., 2022). These modifications result in the development of the VR-12 peptide. Raman and CD spectroscopic analyses confirmed that the structural changes contributed to the antibacterial efficacy of the peptide.

Meanwhile, assessments such as MIC, MBC, and biofilm susceptibility assay have demonstrated the strong antibacterial efficacy of VR-12. SEM analysis revealed that VR-12 destroyed the bacterial cell membrane, leading to bacterial shrinkage and rupture. CLSM confirmed VR-12’s ability to remove mature bacterial biofilms. Furthermore, VR-12 exhibited favorable biocompatibility and hemocompatibility at effective antibacterial concentrations. VR-12 was further observed to promote HGF migration by wound healing and Transwell assays. Ultimately, RT-qPCR analysis indicated that VR-12 upregulates the expression of migration-related genes, FNⅠ and FAK, in HGFs. Therefore, VR-12 not only promotes the proliferation of HGFs but also potentially facilitates their migration by upregulating the expression of migration-related genes, thereby contributing to wound healing.

Macrophages play various essential roles in the host immunity and in maintaining homeostasis in the body, serving as the primary defense line of the immune system (Li et al., 2021). The results of this study indicated that VR-12 could promote the proliferation of RAW264.7 cells at a certain concentration. Given that immune cell proliferation can reflect the organism’s immune status, VR-12 may positively modulate immunity by influencing RAW264.7 cell proliferation. Moreover, VR-12 exhibited the ability to reduce the relative levels of ROS in LPS-stimulated RAW264.7 cells, suggesting its potential as an antioxidant with a certain degree of inhibitory effect on oxidative stress damage induced in these cells.

The RT-qPCR findings demonstrated that treatment with VR-12 resulted in a decrease in the expression of pro-inflammatory factors like CD86 while boosting the presence of anti-inflammatory factors such as CD206 in RAW264.7 cells under inflammatory conditions induced by LPS. This suggests that VR-12 mitigates the pro-inflammatory polarization of macrophages and alleviates the inflammatory response. ELISA analysis further confirmed that VR-12 suppressed LPS-induced TNF-α secretion while enhancing IL-10 secretion in RAW264.7 cells, consistent with mRNA expression patterns, indicating that VR-12 may penetrate macrophages to stimulate cytokine secretion and immune response activation. The observed anti-inflammatory effects of VR-12 are speculated to involve signaling pathways like JAK-STAT3 and NF-κB. These results of peptide modification suggest that short peptides rich in tryptophan and tyrosine play an effective role in maintaining the peri-implantitis inflammatory response. It has potential effects in the management of peri-implantitis. However, this study was limited by the lack of a detailed analysis of the anti-inflammatory mechanism.

## 5 Conclusion

VR-12 was designed based on the antimicrobial peptide IDR-1018. VR-12 significantly inhibited the main pathogens of peri-implantitis in both the planktonic and biofilm states. Furthermore, VR-12 enhanced the proliferation and migration of HGFs and upregulated the expression of genes associated with cell migration to facilitate soft tissue healing. Additionally, VR-12 displayed favorable immunomodulatory characteristics. Nevertheless, the efficacy of this antimicrobial peptide in animal models was not confirmed in this study and a more in-depth exploration of the precise mechanism of action of VR-12 is warranted.

## Data Availability

The datasets presented in this study can be found in online repositories. The names of the repository/repositories and accession number(s) can be found in the article/Supplementary Material.

## Abbreviations

AMPs, Antimicrobial peptides; Arg-1, arginase-1; ANOVA, A one-way analysis of variance; AO/EB, Acridine orange/ethidium bromide; CD, Circular dichroism spectroscopy; CLSM, Confocal laser scanning microscopy; CCK-8, Cell counting kit-8; FNⅠ, Fibronectin Ⅰ; FAK, Focal adhesion kinase; FBS, Fetal bovine serum; GAPDH, Glyceraldehyde-3-phosphate dehydrogenase; HGFs, Human gingival fibroblasts; HDPs, Host defense peptides; HPLC, High-performance liquid chromatography; HR, Hemolysis rate; IDRs, Innate defense regulators; INOS, Inducible nitric oxide synthase; IL-10, interleukin-10; LPS, lipopolysaccharide; MS, Mass spectrometry; MW, Molecular weight; MIC, Minimum inhibitory concentration; MBC, Minimum bactericidal concentration; PBS, Phosphate-buffered saline; PI, Propidium iodide solution; RT-qPCR, Quantitative reverse transcription-polymerase chain reaction; ROS, Reactive oxygen species; SEM, Scanning electron microscopy; SD, Standard deviation; TNF-a, Tumor necrosis factor-alpha.
