# Supplementary material for: Study of an arginine- and tryptophan-rich antimicrobial peptide in peri-implantitis
Source: Front Bioeng Biotechnol. 2025 Jan 7;12:1486213. doi: 10.3389/fbioe.2024.1486213 (PMC11747041; doi:10.3389/fbioe.2024.1486213)
Supplement: Supplementary file 1 [file Table1.docx]

**Supplementary Information**

**Table S1**. Design of cell migration related genes primer.

| **Gene** | **Forward Primer Sequence (5’ -3’ )** | **Reverse Primer Sequence(5’-3’)** |
| --- | --- | --- |
| FAK | GAAGCCTTGCCAGCCTCA | GTGGGGCTGGCTGGATTT |
| FnⅠ | GTCAGCCCAACTCCCACC | TTGGTGGCCGTACTGCTG |
| GAPDH | GCACCGTCAAGGCTGAGAAC | TGGTGAAGACGCCAGTGGA |

**Table S2**. Primer sequences for inflammation-related genes.

| **Gene** | **Forward Primer Sequence (5’ -3’ )** | **Reverse Primer Sequence(5’-3’)** |
| --- | --- | --- |
| iNOS | GAGACAGGGAAGTCTGAAGCAC | CCAGCAGTAGTTGCTCCTCTTC |
| TNF-α | GGTGCCTATGTCTCAGCCTCTT | GCCATAGAACTGATGAGAGGGAG |
| CD86 | ACGTATTGGAAGGAGATTACAGCT | TCTGTCAGCGTTACTATCCCGC |
| IL-10 | CAGTCGGCCAGAGCCACAT | CTTGGCAACCCAAGTAACCCTT |
| Arg-1 | CATTGGCTTGCGAGACGTAGAC | GCTGAAGGTCTCTTCCATCACC |
| CD206 | GTTCACCTGGAGTGATGGTTCTC | GTTCACCTGGAGTGATGGTTCTC |
| GAPDH | CATCACTGCCACCCAGAAGACTG | ATGCCAGTGAGCTTCCCGTTCAG |
